# Blockade of KAT II Facilitates LTP in Kynurenine 3-Monooxygenase Depleted Mice

**DOI:** 10.1177/11786469211041368

**Published:** 2021-08-30

**Authors:** Sophie Imbeault, Max Gubert Olivé, Oscar Jungholm, Sophie Erhardt, Holger Wigström, Göran Engberg, Kent Jardemark

**Affiliations:** 1 Department of Physiology and Pharmacology, Karolinska Institutet, Stockholm, Sweden; 2 Department of Medical Biophysics, Institute of Neuroscience and Physiology, University of Gothenburg, Sweden

**Keywords:** Kynurenine, hippocampus, electrophysiology, long-term potentiation, spatial memory

## Abstract

Excess of brain kynurenic acid (KYNA), a neuroactive metabolite of the kynurenine pathway, is known to elicit cognitive dysfunction. In the present study, we investigated spatial working memory in mice with elevated levels of KYNA, induced by targeted deletion of kynurenine 3-monooxygenase (KMO), as well as long-term potentiation (LTP) of field excitatory postsynaptic potentials (fEPSPs) in hippocampal brain slices from these mice. The KMO knock-out (KMO^−/−^) mice performed more poorly in the spatial working memory task as compared to their wild-type (WT) counterparts, as reflected by fewer correct choices in a T-maze. Both fEPSPs, or LTP, did not significantly differ between the 2 mouse strains. However, administration of PF-04859989, a kynurenine aminotransferase (KAT) II inhibitor, limiting the production of KYNA, facilitated fEPSP and enhanced LTP to a greater extent in hippocampal slices from KMO^−/−^ mice compared to WT mice. The results of the present study point to an essential role for KYNA in modulating LTP in the hippocampus of KMO^−/−^ mice which may account for their dysfunctional spatial working memory.

## Introduction

**Figure 1.**
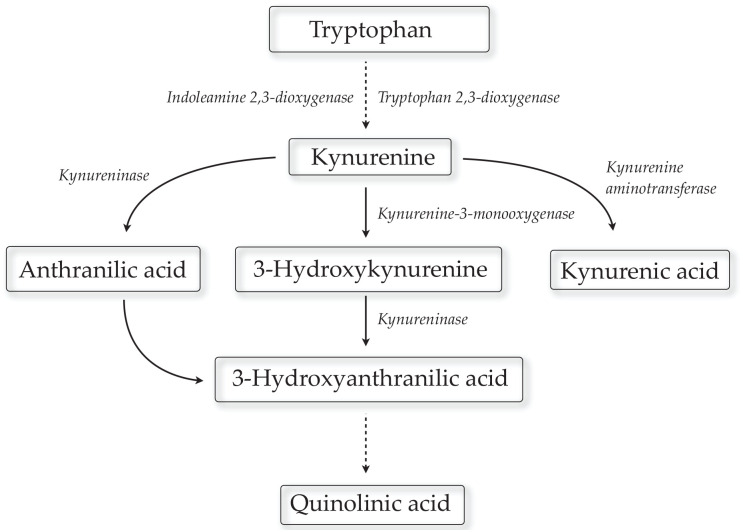
Simplified illustration of the kynurenine pathway. Kynurenine-3-monooxygenase (KMO) deficiency will increase the availability of kynurenine to facilitate KYNA synthesis by the transamination process.

Tryptophan degradation along the kynurenine pathway (Figure 1) has become fundamental in contemporary thoughts about the pathophysiology of psychiatric illnesses. The pathway is induced by immune activation and yields several neuroactive compounds,^1-5^ the most salient being kynurenic acid (KYNA). This metabolite is an antagonist at the glycine site of the N-methyl-D-aspartate (NMDA) receptor and the α-7 nicotinic acetylcholine receptor.^2,6^ Increased central levels of KYNA, are found in patients with schizophrenia and as well as in bipolar disorder patients with a history of psychosis.^4,7-14^

A metabolic step essential for the formation of KYNA is the transamination of kynurenine by kynurenine aminotransferase (KAT) II.^2^ In addition, kynurenine 3-monooxygenase (KMO), which enables the formation of several downstream metabolites, including the glutamate agonist quinolinic acid (QUIN), indirectly controls the production of KYNA. Thus, inhibition of this enzyme will increase the availability of kynurenine to produce KYNA by the transamination process.^2,15^ Accordingly, mice with a targeted genomic deletion of KMO show elevated brain KYNA levels.^5,16,17^ Experimental studies show that increasing brain KYNA by pharmacological or genetic manipulation is associated with behavioral aberrations, including disruption in prepulse inhibition,^18,19^ enhanced amphetamine-induced locomotor activity,^5,13,20^ working memory deficits,^21^ and disruptions in hippocampus-mediated learning and memory in rodents.^22-24^ Indeed, alterations in hippocampal anatomy, perfusion, and activation (eg, impairments in declarative memory function) are consistently reported in schizophrenia.^25^ In addition, long-term-potentiation (LTP) in the hippocampus, a phenomenon that is generally agreed to underlie learning and memory processes, is disrupted in KMO knock-out (KMO^−/−^) mice or mice treated with kynurenine, the immediate precursor of KYNA.^26^ Notably, there are also a number of clinical studies supporting a role for KYNA in cognition and elevated brain KYNA is shown to underlie impairment of cognitive functions in bipolar disorder,^4^ Herpes Simplex encephalitis,^27^ and cerebral malaria.^28^

We reported recently that KMO^−/−^ mice show impaired hippocampus-dependent contextual memory, deficits in social interaction, and anxiety-like behavior.^29^ In the present study, we analyze if deficiency in spatial working memory in these mice is specifically associated with changes in hippocampal long-term potentiation as induced by elevated brain KYNA.

## Materials and Methods

### Animals

Adult male KMO^−/−^ and wild-type (WT) FVB/N mice aged 12 to 17 weeks were separately group-housed (n = 2-6) under standard laboratory conditions in a temperature-controlled environment. Animals were bred in the same room as distinct colonies because maternal KMO genotype may affect kynurenine pathway metabolite production in the offspring.^30^ The animals were kept on a 12 hour light-dark cycle (lights on at 06:00) with food and water available ad libitum. For animals undergoing behavioral testing, food restriction to maintain 85% to 90% of free feeding body weight was initiated at least 3 days prior to the start of testing and maintained throughout the test. The food reward was also introduced at this point to avoid neophagia and consisted of chocolate flavored rice puffs (Coco Pops, Kellogg’s). Ad libitum feeding was reinstated following testing. Animals were handled by the experimenter for 1 week prior to the start of behavioral experiments. The animal procedures were approved by and performed in accordance with the guidelines of the Ethical Committee of Northern Stockholm (N55/14) and those of Directive 2010/63/EU.

### Rewarded alternations in the T-maze

The T-maze apparatus (T-maze, Harvard Apparatus) was modified for use with FVB/N mice, who develop blindness, to include both visual and tactile stimuli. The T-maze protocol followed that of Deacon and Rawlins.^31^ On day 1, animals receive group habituation where up to 5 animals were placed in the T-maze for 10 minutes and receive unlimited rewards once the previous reward had been eaten from the food cup. On days 2 to 4, each animal received 10 minutes of training where 1 arm was blocked and the animal had to consume the reward before gently guided to the starting point. Alternating left-right trials were given. On day 5, each animal received 3 trials of the test procedure where a sample run was first given (with 1 arm blocked) and then followed by a choice run where the animal had to choose whether to go left or right. A correct choice was noted when the animal was heading into the arm that still contained the reward. A choice was considered to be made once the tail tip had entered the arm. The delay between the sample run and the free run was 10 seconds while the inter-trial interval was 10 minutes. The choice of the blocked arm (right or left) was randomly assigned for each trial.

### Preparation of hippocampal slices from mice

Mice were anesthetized with isoflurane (Abbot Laboratories Ltd, UK) and decapitated. The brain was removed and placed in an ice-cold oxygenated (95% O_2_/5% CO_2_) Ringer’s solution containing (in mM): 126 NaCl, 2.5 KCl, 1.2 NaH_2_PO_4_, 1.3 MgCl_2_, 2.4 CaCl_2_, 18 NaHCO_3_, and 10 D-glucose; pH 7.4. The hippocampus was isolated and 400 µm thick transverse slices were cut using a Starret 263M-tissue chopper. The slices were then kept in Ringer’s solution for a recovery period of minimum 1 hour at room temperature before being transferred to the recording chamber.

### Electrophysiological recording of fEPSPs and LTP in hippocampal slices

Hippocampal slices from the KMO^−/−^ or WT mice, were continuously superfused with oxygenated Ringer’s solution at a rate of 4 mL/min at 30°C. Field excitatory postsynaptic potentials (fEPSP) were evoked by electrically stimulating the Schaffer collateral-commissural fibers, and recorded in *stratum radiatum* of the hippocampal CA1 region. A monopolar tungsten electrode positioned in the same layer was used for stimulation, delivering negative constant-current pulses with a duration of 0.3 ms and amplitude of 40 to 70 μA. The extracellular recordings were made via a borosilicate glass micropipette (ie, 0.58 mm, 3-5 MΩ) filled with 2M NaCl mixed with Chicago Sky Blue dye to improve visibility. Once a stable fEPSP was found, a 10 minutes period was recorded as a baseline.

LTP was induced by theta-burst stimulation (TBS), consisting of 3 trains of 10 pulses at 100 Hz, repeated with a burst frequency of 5 Hz. Three such sets of stimuli were given, normally separated by 10 seconds. Drugs were applied via bath perfusion and a period of 30 minutes was allowed for incubation before LTP induction. The recordings were acquired with the in-house designed hardware and software equipment Biomux.

### Data analysis

Behavioral data were analyzed using repeated-measures two-way ANOVA followed by post-hoc LSD, unpaired *t*-test, or a one sample *t*-test with a theoretical mean (to test whether groups performed differently than chance). Offline data analysis was performed using pCLAMP-Clampfit 10 (Molecular Devices, USA). The effect of LTP was quantified by measuring the initial slope of the evoked fEPSP and comparing it to the initial baseline prior to theta-burst stimulation. Statistical analysis was performed using GraphPad Prism 7 (GraphPad software, USA). Electrophysiological data were analyzed using unpaired *t*-test. Data was presented as mean ± standard error of the mean, and *P* values <.05 were considered significant.

### Drugs and chemicals

In order to obtain stock solutions, PF-04859989 hydrochloride (Sigma-Aldrich, USA) was dissolved in purified water. The compound was dissolved to their final concentrations in Ringer’s solution.

## Results

### Spatial working memory assessment in WT and KMO^−/−^ mice

**Figure 2.**
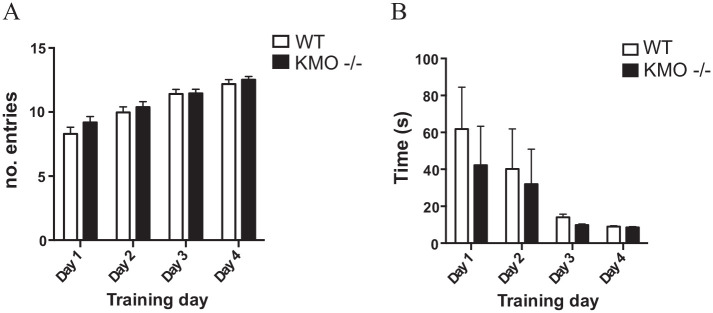
Acquisition learning is similar between KMO^−/−^ and WT mice. (A) Total number of entries into the arms of the T-maze made over the course of training. (B) Latency to complete the first trial of the training session on the indicated training day. Data are mean ± SEM. Two-way repeated measures ANOVA.

**Figure 3.**
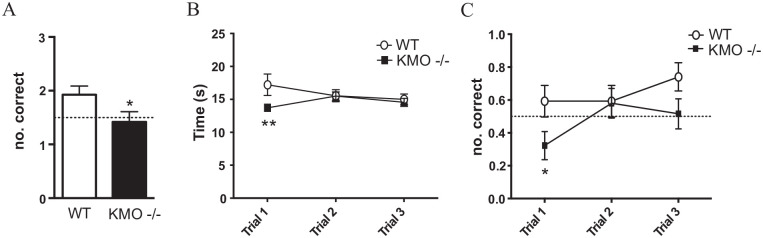
KMO^−/−^ mice exhibit working memory deficits. (A) Number of correct responses in the free run of the rewarded alternations T-maze (*P < .05, *t*-test). (B) Total time to trial completion. (C) Number of correct responses per group over 3 testing trials. Two-way repeated measures ANOVA *P < .05, **P < .01 post-hoc Fisher’s LSD. Data are mean ± SEM; dotted lines indicate chance responding.

Spatial working memory was studied using rewarded alternations in the T-maze. During the acquisition phase, both WT and KMO^−/−^ mice learned to retrieve the food reward in a similar fashion. Both genotypes increased the number of rewards consumed within 10 minutes and decreased the latency to consume the first reward over the 4 training days. There was no significant difference between the genotypes with regard to the number of arm entries (Figure 2A) or the time taken to consume the first reward (Figure 2B). However, when the task was changed to test working memory, KMO^−/−^ mice performed more poorly than WT controls. Overall, KMO^−/−^ mice made fewer correct choices than WT animals (Figure 3A, WT 1.9 ± 0.2, n = 27; KMO^−/−^ 1.4 ± 0.2, n = 31; *P* = .049, *t*-test, dotted line indicating chance response). In fact, while WT animals performed significantly higher than chance (*P* = .013, one-sample *t*-test using chance as the theoretical mean), KMO^−/−^ animals did not (*P* = .67, one-sample *t*-test using chance as the theoretical mean). When broken down over the test trials, there was a significant interaction between genotype and trial number in the time required for trial completion (Figure 2B) (interaction: *F*_(2,112)_ = 4.67, *P* = .011; trial effect: *F*_(2,112)_ = 0.91, *P* = .41; genotype effect: *F*_(1,56)_ = 1.71, *P* = .20) with a significant post-hoc difference in the first trial between knockouts and controls (*P* = .0056, post-hoc LSD). When tracking the number of correct trials (Figure 3C), there was a significant effect of genotype on correct performance over trials (genotype effect: *F*_(1,56)_ = 4.04, *P* = .049; interaction: *F*_(2,112)_ = 1.325, *P* = .27; trial effect: *F*_(2,112)_ = 2.215, *P* = .11) with a significant post-hoc difference for the first trial (*P* = .038, post-hoc LSD). In sum, even though KMO^−/−^ mice took significantly less time than controls to complete the first trial, they were more often wrong.

### LTP generation in the CA1 region of hippocampus in WT and KMO^−/−^ mice

**Figure 4.**
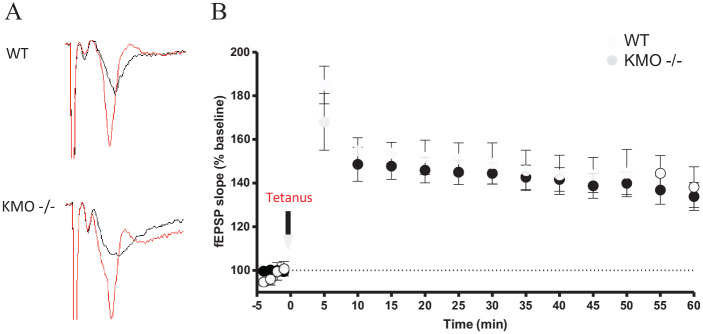
LTP in hippocampal slices of WT and KMO^−/−^ mice. The fEPSP traces from (A) WT and KMO^−/−^ mice show the differences between recordings before theta-burst stimulation (black) and the potentiated response after (red). (B) LTP generation in WT (n = 5) and KMO^−/−^ (n = 5) mice from the CA1 region of the hippocampus. Theta-burst (tetanus) stimulation is indicated by the black arrow. Data is presented as mean ± SEM.

To study alterations in glutamatergic neurotransmission under a condition of KYNA excess, differences in LTP generation in the CA1 region in hippocampal slices from WT and KMO^−/−^ mice were examined (Figure 4A and B). Here, we compared the slope of the evoked fEPSP after theta-burst (tetanus) stimulation between WT (n = 5) and KMO^−/−^ (n = 5) mice. A similar LTP generation profile was obtained in hippocampal slices from the 2 groups but of slightly lower magnitude in the slices from the KMO^−/−^ mice. Although this difference did not attain statistical significance, it is notable that the average values of LTP in KMO^−/−^ slices were smaller than those in WT at all time points (Figure 4B).

### Effects of the KAT II inhibitor PF-04859989 on fEPSPs and LTP generation in hippocampal slices

**Figure 5.**
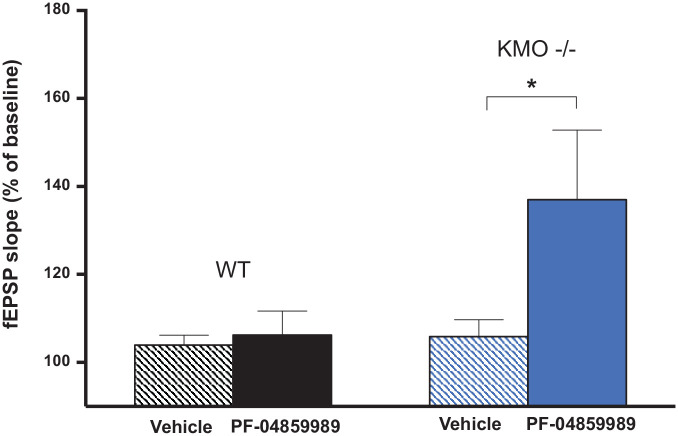
Effects of 1 mM PF-04859989 on the fEPSPs in hippocampus slices from WT and KMO^−/−^ mice. Changes in the fEPSPs during the 30 minutes perfusion time with PF-04859989 or vehicle in hippocampal slices from WT (n = 5) or KMO^−/−^ (n = 5) mice. Data is presented as mean ± SEM; *P < .05, unpaired *t*-test.

**Figure 6.**
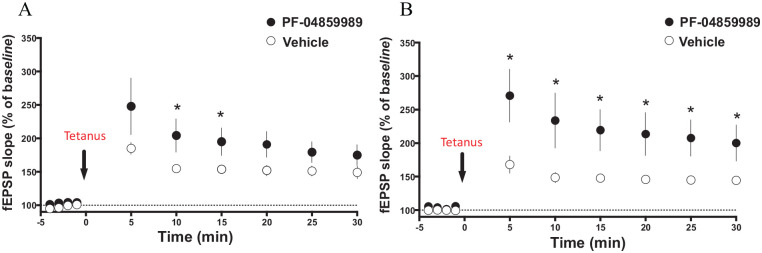
Effects of 1 mM PF-04859989 on LTP generation in hippocampal slices from WT and KMO^−/−^ mice. LTP generation in hippocampal slices from (A) WT and (B) KMO^−/−^ mice, perfused with PF-04859989 compared to drug-naïve conditions (ie, vehicle). Theta-burst (tetanus) stimulation is indicated by the black arrow. Data is presented as mean ± SEM; *P < .05, unpaired *t*-test.

Next, we studied the effects of the KAT II inhibitor PF-04859989 on fEPSPs and LTP generation in hippocampal slices from KMO^−/−^ and WT mice, respectively. We perfused the hippocampal slices with PF-04859989 in a concentration of 1 µM^32^ for 30 minutes prior to the induction of LTP. PF-04859989 significantly facilitated the fEPSP slope in the hippocampal slices from the KMO^−/−^ mice (Figure 5; n = 5; *P* < .05, unpaired *t*-test). Interestingly, PF-04859989 did not affect fEPSPs in slices from the WT mice (Figure 5; n = 5). After theta-burst stimulation, the LTP was significantly facilitated by PF-04589989 in the hippocampal slices from the WT mice, after 10 minutes of drug administration (Figure 6A; *P* < .05, unpaired *t*-test; n = 5), and this effect disappeared after 15 minutes. PF-04859989 also facilitated LTP generated in hippocampal slices from the KMO^−/−^ mice, but already after 5 minutes (Figure 6B, *P* < .05, unpaired *t*-test; n = 5) of drug administration, and the effect sustained more than 30 minutes.

## Discussion

This study examined spatial working memory, glutamatergic neurotransmission, and LTP generation in the CA1 region of hippocampal slices in KMO^−/−^ mice with altered extracellular KYNA concentration. We found that KMO^−/−^ mice show deficits in spatial working memory and that hippocampal excitability and LTP in these mice are related to KYNA synthesis. Moreover, the KAT II inhibitor PF-04859989 facilitated glutamate receptor-mediated fEPSPs in hippocampal slices from KMO^−/−^ mice, but not from WT mice. PF-04859989 enhanced LTP in hippocampal slices from both KMO^−/−^ and WT mice, but the effect of this drug on LTP in the slices from the KMO^−/−^ mice was faster and sustained over 30 minutes.

Here, we tested spatial working memory, which depends on the hippocampus,^33^ using the rewarded alternations paradigm in the T-maze. During the acquisition phase, both genotypes showed a similar ability and motivation to retrieve the food reward, as shown by the increased number of rewards consumed and decreased latency to consume the first reward. It was only when the task was switched to engage working memory, defined in this case as the short-term retention of information from the sample run, in order to correctly complete the free run, that KMO^−/−^ mice began to perform poorly. The fact that KMO^−/−^ mice performed no differently than chance suggests they have not properly retained the information of which arm was first entered and are therefore essentially guessing which arm to enter during the free run. Upon closer examination, significant deficits in performance could be seen in the first trial of the testing block, whereby KMO^−/−^ mice made faster choices than WT mice but were more often incorrect. Our results are also consistent with other tests performed in rodents showing elevated KYNA levels impair cognitive performance.^5,21,34^ It has previously been shown that infusion of 2-amino-5-phosphonopentanoic acid (AP5), a competitive NMDAR antagonist known to disrupt LTP,^35,36^ also produces impairment in the rewarded T-maze alternation paradigm.^37,38^ Although this result did not reach statistical significance, perhaps due to small numbers, the trend toward impaired LTP in hippocampal slices from KMO^−/−^ mice observed here is in line with the poor performance observed in the T-maze. The amount of delay between the choice and sample runs was determined in pilot experiments whereby the delay was set to allow wild-type animals to perform at approximately 70% correct responses, thus allowing detection of impairments and improvements across all our experiments with FVB/N mice. It is possible that with a shorter (or no) delay, KMO^−/−^ mice would not show cognitive impairment and that conversely, more pronounced issues would be observed with a longer delay. Furthermore, although the dorsal hippocampus is crucial to working memory paradigms with a spatial component, other brain regions are also involved.^31^ Notably, the prefrontal cortex, which is also associated with attentional processes, has been shown to be specifically involved in spatial memory processes.^39^ Therefore, given the results of the LTP experiments in the present study, it is also possible alterations in circuits outside of the hippocampus contribute to the observed working memory deficits.

LTP in neurons of hippocampus reflects strengthening glutamatergic synapses in an activity-dependent manner, a process that requires activation of postsynaptic NMDA receptors and associated Ca^2+^ influx as the initial step. The following intracellular events triggered by the Ca^2+^ influx, leading up to the synaptic strengthening process, are primarily dependent on the recruitment of AMPA receptors.^40^ Since KYNA can negatively modulate NMDA receptors at the glycine site,^41-44^ variations in KYNA concentration provides a means to interfere with LTP generation. In support of this idea, previous studies in transgenic mice with over-production of KYNA revealed a decreased ability for LTP.^26^ Our present results are consistent with those findings, although fEPSP or LTP was not significantly different between WT mice and KMO^−/−^ mice. Notably, at higher concentrations KYNA also competitively blocks the AMPA receptor.^43,45^ Thus, although the glycine site of the NMDA receptor may be a preferable site of action by KYNA to influence hippocampal LTP, we cannot exclude non-NMDA receptor involvement, since the postsynaptic AMPA receptors, in particular, are critically involved in the LTP process.^46^

To further elucidate the role of KYNA in LTP, pharmacological manipulation of endogenous KYNA levels was performed utilizing administration of the KAT II inhibitor PF-04859989. Considering that increased endogenous KYNA will lead to impaired LTP generation,^26^ 1 would expect the opposite to take place with lowered KYNA concentration. This idea was tested in the present study, assessing LTP in slices treated with the KAT II inhibitor PF-04859989. Adding this inhibitor will interfere with the enzymatic output stage of KYNA production,^47-49^ leading to lowering of KYNA and thereby facilitation of NMDA receptors. Indeed, previous results have indicated that KAT II-deficient mice show an increase in the amplitude of LTP in the hippocampus compared to WT controls.^50^ In the present study, we found that PF-04859989 facilitated the fEPSPs in hippocampal slices from KMO^−/−^ mice, but not from WT mice. PF-04859989 also produced a faster and more sustained facilitating effect on LTP in the slices from the KMO^−/−^ mice, compared with the effect in the slices from the WT mice. The lack of effect by PF-04859989 on fEPSPs, as well as on the moderate effect on LTP, in slices from WT mice is puzzling but may result from compensatory changes of NMDA receptor expression or functioning, tentatively occurring in the KMO^−/−^ mice. Such a condition, involving also decreased KYNA concentration, as induced by the KAT II inhibitor, may generate a most favorable state for LTP.

Previous studies in the rat clearly show that endogenous brain KYNA tonically control the firing rate of rat midbrain dopamine neurons.^49,51^ However, as fEPSPs were not affected by PF-04859989 perfusion in the WT mice, endogenous KYNA in these mice may not tonically affect glutamatergic transmission, in contrast to its regulatory actions in rats, Alternatively, the CA1 region of the hippocampus may differ from midbrain dopamine neurons in this regard.

It should be noted that the estrous cycle has been shown to influence spontaneous alternation behavior^52^ so we limited our study to male mice. While differences in tryptophan and kynurenine pathway metabolites exist in the periphery between human males and females^53^ the data concerning KYNA levels in the central nervous system are not as clear cut,^4,54^ while mouse brain kynurenic acid levels appear similar between genders.^55^ Nonetheless, and given differences in pharmacokinetics, it could be interesting to investigate the effect of PF-04859989 on learning and memory processes in female subjects either using alternation behavior with control for the estrous cycle or another type of memory task.

In conclusion, the results of the present study demonstrate that a genetic model with overproduction of brain KYNA shows spatial working memory dysfunction. Although no significant differences in hippocampal fEPSP or LTP were observed between KMO^−/−^ mice and WT mice, inhibition of KAT II, decreasing endogenous KYNA levels, was associated with a faster and more sustained facilitating effect on LTP in KMO^−/−^ mice, compared to WT mice. Our results indicate an essential role of KYNA in the regulation of LTP and, in its functional extension, cognitive functions.
